# Characterization of Membrane Potential Dependency of Mitochondrial Ca^2+^ Uptake by an Improved Biophysical Model of Mitochondrial Ca^2+^ Uniporter

**DOI:** 10.1371/journal.pone.0013278

**Published:** 2010-10-08

**Authors:** Ranjan K. Pradhan, Feng Qi, Daniel A. Beard, Ranjan K. Dash

**Affiliations:** Biotechnology and Bioengineering Center and Department of Physiology, Medical College of Wisconsin, Milwaukee, Wisconsin; German Cancer Research Center, Germany

## Abstract

Mitochondrial Ca^2+^ uniporter is the primary influx pathway for Ca^2+^ into respiring mitochondria, and hence plays a key role in mitochondrial Ca^2+^ homeostasis. Though the mechanism of extra-matrix Ca^2+^ dependency of mitochondrial Ca^2+^ uptake has been well characterized both experimentally and mathematically, the mechanism of membrane potential (ΔΨ) dependency of mitochondrial Ca^2+^ uptake has not been completely characterized. In this paper, we perform a quantitative reevaluation of a previous biophysical model of mitochondrial Ca^2+^ uniporter that characterized the possible mechanism of ΔΨ dependency of mitochondrial Ca^2+^ uptake. Based on a model simulation analysis, we show that model predictions with a variant assumption (Case 2: external and internal Ca^2+^ binding constants for the uniporter are distinct), that provides the best possible description of the ΔΨ dependency, are highly sensitive to variation in matrix [Ca^2+^], indicating limitations in the variant assumption (Case 2) in providing physiologically plausible description of the observed ΔΨ dependency. This sensitivity is attributed to negative estimate of a biophysical parameter that characterizes binding of internal Ca^2+^ to the uniporter. Reparameterization of the model with additional nonnengativity constraints on the biophysical parameters showed that the two variant assumptions (Case 1 and Case 2) are indistinguishable, indicating that the external and internal Ca^2+^ binding constants for the uniporter may be equal (Case 1). The model predictions in this case are insensitive to variation in matrix [Ca^2+^] but do not match the ΔΨ dependent data in the domain ΔΨ≤120 mV. To effectively characterize this ΔΨ dependency, we reformulate the ΔΨ dependencies of the rate constants of Ca^2+^ translocation via the uniporter by exclusively redefining the biophysical parameters associated with the free-energy barrier of Ca^2+^ translocation based on a generalized, non-linear Goldman-Hodgkin-Katz formulation. This alternate uniporter model has all the characteristics of the previous uniporter model and is also able to characterize the possible mechanisms of both the extra-matrix Ca^2+^ and ΔΨ dependencies of mitochondrial Ca^2+^ uptake. In addition, the model is insensitive to variation in matrix [Ca^2+^], predicting relatively stable physiological operation. The model is critical in developing mechanistic, integrated models of mitochondrial bioenergetics and Ca^2+^ handling.

## Introduction

Mitochondrial Ca^2+^ uniporter is the primary influx pathway for Ca^2+^ into respiring mitochondria, and hence is a key regulator of mitochondrial Ca^2+^. Mitochondrial Ca^2+^ homeostasis is critical for metabolic regulation, mitochondrial function/dysfunction, and cell physiology/pathophysiology [1]–[9]. Therefore, a mechanistic characterization of mitochondrial Ca^2+^ uptake via the uniporter is essential for developing mechanistic, integrated models of mitochondrial bioenergetics and Ca^2+^ handling that can be helpful in understanding the mechanisms by which Ca^2+^ plays a role in mediating signaling pathways between cytosol and mitochondria and modulating mitochondrial energy metabolism in health and disease [10], [11].

The kinetics of mitochondrial Ca^2+^ uptake depends on the catalytic properties of the uniporter and also on the electrochemical gradient of Ca^2+^ across the inner mitochondrial membrane (IMM), which has been extensively studied both experimentally [12]–[18] and with the help of mathematical models [10], [11], [19]–[21]. Though the mechanism of extra-matrix Ca^2+^ dependency of mitochondrial Ca^2+^ uptake has been well characterized, the mechanism of membrane potential (ΔΨ) dependency of mitochondrial Ca^2+^ uptake has not been completely characterized.

In a recent paper [11], we introduced a mechanistic mathematical model of mitochondrial Ca^2+^ uniporter (presented briefly in Materials S1) that satisfactorily describes the available experimental data on the kinetics of mitochondrial Ca^2+^ uptake, measured in suspensions of respiring mitochondria isolated from rat hearts and rat livers under various experimental conditions [12], [13], [16]. This model is developed based on a multi-state catalytic binding and interconversion mechanism (Michaelis-Menten kinetics) for carrier-mediated facilitated transport [22], [23], and Eyring's free-energy barrier theory for interconversion and electrodiffusion [22], [24]–[26]. The model also accounts for possible allosteric, cooperative binding of Ca^2+^ to the uniporter, as seen experimentally [12], [13]. Therefore, the biophysical formulation, thermodynamic feasibility, and ability to explain a large number of independent experimental data sets are some of the remarkable features of the model [11], compared to the previous models of the uniporter [19]–[21]. The model was able to characterize the possible mechanisms of both the extra-matrix Ca^2+^ and ΔΨ dependencies of the uniporter-mediated mitochondrial Ca^2+^ uptake [12], [13], [16].

In the development of our recent model of the uniporter [11], two different kinetic models (Model 1 or Model 2: fully or partial cooperativity of Ca^2+^ binding to the uniporter) under two different kinetic assumptions (Case 1 or Case 2: external and internal Ca^2+^ binding constants for the uniporter are equal or distinct) were formulated to characterize the extra-matrix Ca^2+^ and ΔΨ dependencies of mitochondrial Ca^2+^ uptake via the uniporter [12], [13], [16] (see Materials S1). Both the models under both the cases were able to satisfactorily describe the extra-matrix Ca^2+^ dependent data [12], [13]. However, the models under two different cases provided two significantly different predictions of the ΔΨ dependent data [16], especially in the domain ΔΨ≤120 mV. While the models under Case 1 were not able to simulate the ΔΨ dependent data in the domain ΔΨ≤120 mV, the models under Case 2 were able to satisfactorily reproduce the ΔΨ dependent data in the entire ΔΨ domain for which data were available. Based on these kinetic analyses, Case 2 was determined to be the most plausible representation of the observed ΔΨ dependency of the uniporter-mediated mitochondrial Ca^2+^ uptake.

The four variant models of the uniporter [11] were parameterized exclusively based on the experimental data [12], [13], [16] in which matrix [Ca^2+^] was unknown from the measurements. For model parameterization, matrix [Ca^2+^] was fixed at 250 nM. Although the two variant models under Case 2 were able to adequately describe all the available experimental data with appropriate model perturbations as provided by the experimental protocols, it was unknown whether physiological variation of matrix [Ca^2+^], as seen in the intact myocyte, have significant impacts on the estimates of model parameters and model predicted trans-matrix Ca^2+^ fluxes via the uniporter. Therefore, it is important to test the robustness of the estimates of model parameters and model predictions subject to such physiological variation.

In the present paper, we attempt to provide a quantitative reevaluation of our previous model of the uniporter [11]. Based on a model simulation analysis, we show that the two variant model predictions under Case 2 are highly sensitive to variation in matrix [Ca^2+^] (ranging from 100 nM to 500 nM), suggesting that the model parameter estimates under Case 2 would vary significantly to variation in matrix [Ca^2+^], and hence can not be robust. This indeed indicates that the Case 2, in which the Ca^2+^ binding constants for the uniporter at the inside and outside of the IMM are distinct, is physiologically implausible, and hence can not be a feasible representation of the observed ΔΨ dependency of the uniporter-mediated mitochondrial Ca^2+^ uptake. Furthermore, the Case 2 is associated with negative estimates of the biophysical parameter *α*_x_ (with *α*_e_ = 0 fixed) (see Table S1), which is found to be contributing to the high sensitivities of the model predictions to variation in matrix [Ca^2+^]. To reconcile this issue, we reestimate model parameters subject to the constraint: *α*_e_ = *α*_x_ = *α*≥0, which implies that the Ca^2+^ binding sites on the uniporter are located at equal distances from the bulk phase on either side of the IMM. This reparameterization shows that the two variant assumptions on the Ca^2+^ binding to the uniporter (Case 1 and Case 2) are indistinguishable from each other, indicating that the external and internal Ca^2+^ binding constants for the uniporter may be equal (Case 1). The model predictions in this case are insensitive to variation in matrix [Ca^2+^], but do not match the ΔΨ dependent data [16] in the domain ΔΨ≤120 mV.

To accurately characterize the ΔΨ dependency of mitochondrial Ca^2+^ uptake via the uniporter in the entire ΔΨ domain for which data are available [16], we reformulate the ΔΨ dependencies of the rate constants *k*_in_ and *k*_out_ of Ca^2+^ translocation in our previous model of the uniporter [11] by exclusively redefining the biophysical parameters *β*_e_ and *β*_x_ associated with the free-energy barrier of Ca^2+^ translocation based on a generalized, non-linear GHK (Goldman-Hodgkin-Katz) formalism (see Materials and Methods). This alternative uniporter model has all the characteristics of our previous uniporter model [11], and is also able to satisfactorily characterize the possible mechanisms of both the extra-matrix Ca^2+^ and ΔΨ dependencies of the uniporter-mediated mitochondrial Ca^2+^ uptake [12], [13], [16]. Furthermore, the model is relatively insensitive to variation in matrix [Ca^2+^], making the model physiologically plausible.

## Results

This section presents the detailed simulation analyses of our previous model of mitochondrial Ca^2+^ uniporter [11] that describe the sensitivity of the model predicted mitochondrial Ca^2+^ uptake in response to physiologically realistic variation in matrix [Ca^2+^] and is used to test the robustness of the estimates of model parameters and model predictions. This section also presents the reparameterization of our previous model of the uniporter [11] and parameterization of the present alternate model of the uniporter subject to the constraint: *α*_e_ = *α*_x_ = *α*≥0 based on the experimental data of Scarpa and coworkers [12], [13] and Gunter and coworkers [16] on the kinetics of Ca^2+^ fluxes via the uniporter. For the purpose of illustrations, only the fully cooperativity binding model (Model 1) under both the kinetic assumptions (Case 1 and Case 2) is chosen, because both the fully and partial cooperativity binding models (Model 1 and Model 2) are indistinguishable from the available experimental data [12], [13], [16] (see Dash et al. [11]).

**Figure 1:**
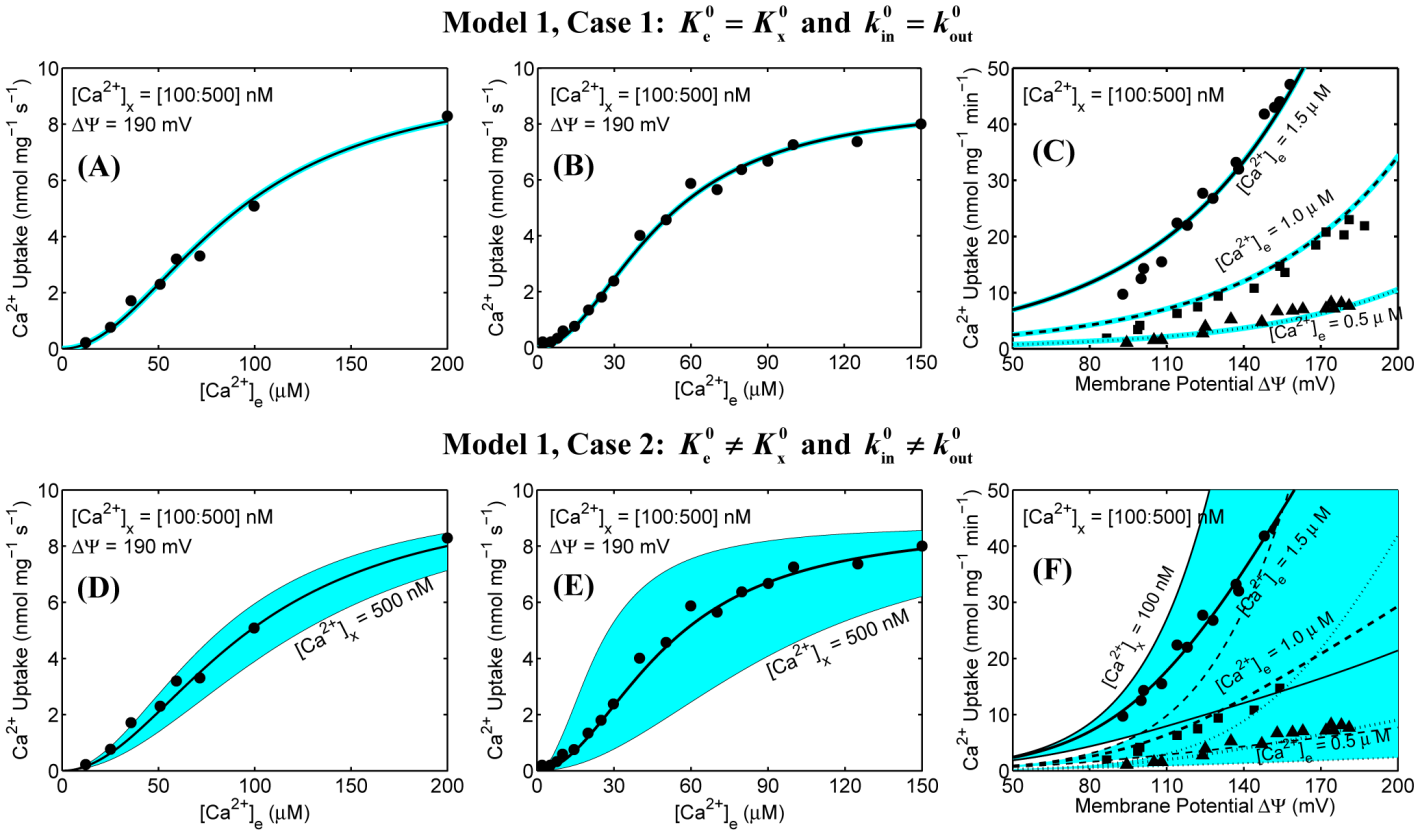
Predicted sensitivities (color maps) of mitochondrial Ca^2+^ uptakes as functions of extra-matrix [Ca^2+^] and ΔΨ in response to variation in matrix [Ca^2+^] using our previous model of mitochondrial Ca^2+^ uniporter and their comparisons to the available experimental data (points). Simulations and fittings are shown only for Model 1 under two different cases (Case 1: upper panel and Case 2: lower panel). The left panel (A and D) shows the simulations and fittings of the model to the kinetic data of Scarpa and Graziotti [12] in which the initial rates of Ca^2+^ uptake were measured in respiring mitochondria isolated from rat hearts with varying levels of extra-matrix Ca^2+^. The middle panel (B and E) shows the simulations and fittings of the model to the kinetic data of Vinogradov and Scarpa [13] in which the initial rates of Ca^2+^ uptake were measured in respiring mitochondria isolated from rat livers with varying levels of extra-matrix Ca^2+^. For these analyses, ΔΨ was set at 190 mV, corresponding to state 2 respiration. The right panel (C and F) shows the simulations and fittings of the model to the kinetic data of Wingrove et al. [16] in which the initial rates of Ca^2+^ uptake were measured as a function of ΔΨ in respiring mitochondria isolated from rat livers with three different levels of extra-matrix Ca^2+^ ([Ca^2+^]_e_ = 0.5 µM, 1.0 µM, and 1.5 µM). For sensitivity color maps, matrix [Ca^2+^] was varied from 100 nM to 500 nM. The black lines corresponding to the model fittings to the data are based on 250 nM of matrix [Ca^2+^]. The other black lines define the borders of the color maps. The model uses the same parameter values as estimated before (Table S1).

**Figure 2:**
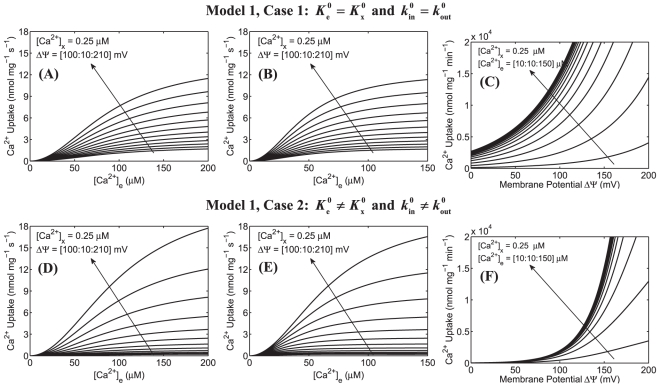
Predictions of mitochondrial Ca^2+^ uptake as a function of extra-matrix [Ca^2+^] for a range of ΔΨ and as a function ΔΨ for a range of extra-matrix [Ca^2+^] based on our previous model of mitochondrial Ca^2+^ uniporter. Simulations are shown only for Model 1 under two different cases (Case 1: upper panel and Case 2: lower panel). The left panel (A and D) shows the model predicted Ca^2+^ uptake in response to varying extra-matrix Ca^2+^ corresponding to the experimental protocol of Scarpa and Graziotti [12] for a range of ΔΨ. The middle panel (B and E) shows the model predicted Ca^2+^ uptake in response to varying extra-matrix Ca^2+^ corresponding to the experimental protocol of Vinogradov and Scarpa [13] for a range of ΔΨ. In these simulations, ΔΨ was varied from 100 mV to 210 mV and matrix [Ca^2+^] was fixed at 0.25 µM. The right panel (C and F) shows the model predicted Ca^2+^ uptake as a function of ΔΨ corresponding to the experimental protocol of Wingrove et al. [16] with extra-matrix [Ca^2+^] ranging from 10 µM to 150 µM and matrix [Ca^2+^] fixed at 0.25 µM. The model uses the same parameter values as estimated earlier (Table S1). The arrows indicate the direction of increasing ΔΨ in plots A, B, D, and E and increasing extra-matrix [Ca^2+^] in plots C and F.

The simulation analyses of mitochondrial Ca^2+^ uptake based on our previous model (Model 1) of mitochondrial Ca^2+^ uniporter [11] are shown in Figures 1 and 2. The upper and lower panels correspond to the simulation analyses for Case 1 and Case 2, while the left, middle, and right panels correspond to the simulation analyses based on the experimental protocols of Scarpa and Graziotti [12], Vinogradov and Scarpa [13], and Wingrove et al. [16], respectively. The model uses the same parameter values as estimated before (see Table S1).

In the experiments of Scarpa and Graziotti [12] and Vinogradov and Scarpa [13], the initial (or pseudo-steady state) rates of Ca^2+^ influx via the uniporter were measured in suspensions of energized mitochondria purified from rat hearts and rat livers following additions of varying levels of extra-matrix Ca^2+^ (with extra-matrix Mg^2+^ fixed at 5 mM and 2 mM, respectively) (Figure 1 (A,D) and 1 (B,E)). In the experiments of Wingrove et al. [16], the initial (or pseudo-steady state) rates of Ca^2+^ influx via the uniporter were measured as a function of ΔΨ in suspensions of energized mitochondria purified from rat livers with three different levels of extra-matrix Ca^2+^ ([Ca^2+^]_e_ = 0.5 µM, 1.0 µM, and 1.5 µM; [Mg^2+^]_e_ = 0 mM) (Figure 1 (C,F)); ΔΨ was varied by adding varying levels of malonate to the extra-matrix buffer medium. In these experiments, matrix [Ca^2+^] was fairly unknown. Our previous model of the uniporter [11] was parameterized based on these experimental data with a fixed matrix [Ca^2+^] of 250 nM. Figure 1 illustrates the effects of physiological variation of matrix [Ca^2+^] on the estimates of model parameters and model predicted Ca^2+^ fluxes via the uniporter.

Specifically, Figure 1 shows the model predicted sensitivities of Ca^2+^ fluxes via the uniporter (lines) as functions of extra-matrix [Ca^2+^] (ΔΨ = 190 mV) and ΔΨ ([Ca^2+^]_e_ = 0.5 µM, 1.0 µM, and 1.5 µM) over a range of matrix [Ca^2+^] along with the experimental data [12], [13], [16] (points). In these simulations, matrix [Ca^2+^] was varied from 100 nM to 500 nM. The simulations corresponding to the model fits to the data are based on matrix [Ca^2+^] of 250 nM. Figure 2 shows the model predicted Ca^2+^ fluxes via the uniporter as a function of extra-matrix [Ca^2+^] for a range of ΔΨ (100 mV to 210 mV) and as a function of ΔΨ for a range of extra-matrix [Ca^2+^] (10 µM to 150 µM), with matrix [Ca^2+^] fixed at 250 nM.

It is apparent from the model simulation analyses in Figure 1 that though the model under Case 2 with matrix [Ca^2+^] fixed at 250 nM is able to fit well to all of the available experimental data [12], [13], [16] with suitable model perturbations as provided by the experimental protocols, the model predictions under this case are extremely sensitive to variation in matrix [Ca^2+^] (Figure 1 (D–F): lower panel). In contrast, the model predictions under Case 1, although do not fit well to the ΔΨ dependent data in the range ΔΨ≤120 mV, are insensitive to variation in matrix [Ca^2+^] (Figure 1 (A–C): upper panel). It is also observed from Figure 2 that the Ca^2+^ uptake profiles under Case 2 have stiff gradients with respect to ΔΨ and reach saturation for a lower level of extra-matrix [Ca^2+^], compared to that under Case 1. Note that the extra-matrix [Ca^2+^] is in µM range, while matrix [Ca^2+^] is in nM range. Therefore, with a positive ΔΨ (ΔΨ = Ψ_e_−Ψ_x_ = outside potential – inside potential; Ψ_e_ is positive and Ψ_x_ is negative) (i.e., with a high electrochemical gradient of Ca^2+^ from the extra-matrix to matrix space), it is unlikely that physiological variation of matrix [Ca^2+^] would have any appreciable effects on the experimental measurements and model predictions on Ca^2+^ fluxes via the uniporter as well as on the estimates of the uniporter model parameters. Furthermore, it is unlikely that in the experiments of Scarpa and colleagues [12], [13] and Gunter and colleagues [16], matrix [Ca^2+^] would have been precisely maintained at 250 nM. Therefore, the present model simulation analyses suggest that the model parameter estimates under Case 2 would vary considerably with variation in matrix [Ca^2+^] as well as with different initial guesses for the parameters, compared to that under Case 1. In the other words, the model parameter estimates under Case 2 would be ambiguous and not unique (robust), and are expected to be different for different matrix [Ca^2+^] and different initial guesses for the parameters. Given matrix [Ca^2+^], the initial guesses for the parameters need to be close to the optimal parameter estimates for the optimization algorithm to converge to the optimal parameter estimates. In this case, the sensitivities of the model to variations in matrix [Ca^2+^] would also be different for different model parameter estimates with similar fittings of the model to the experimental data.

As shown in Table S1, Case 2 is associated with negative estimates of the biophysical parameter *α*_x_ (with *α*_e_ = 0 fixed), which is found to be contributing to the high sensitivities of the model predictions to variation in matrix [Ca^2+^] and stiff gradients of Ca^2+^ uptake profiles to variation in ΔΨ. The Ca^2+^ uptake profiles under Case 2 attaining saturation for a lower level of extra-matrix [Ca^2+^] is attributed to the lower estimates of 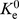 and 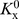 parameters that characterize the binding of [Ca^2+^] to the uniporter (see Table S1). To reconcile this issue, we reestimate our previous uniporter model parameters with an additional constraint: *α*_e_ = *α*_x_ = *α*≥0, an assumption that implies that Ca^2+^ binding sites on the uniporter are located at equal distances from the bulk phase on either side of the IMM. With this constraint, four unknown parameters were estimated for Case 1, while five unknown parameters were estimated for Case 2, using the two kinetic and thermodynamic constraints of Eq. (S7), as in our previous paper on the uniporter [11] (see Materials S1). Here, we follow a three-step modular approach to reparameterize our previous uniporter model. In the first step, the binding constants 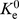 and 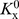 are estimated based on the extra-matrix Ca^2+^ dependent kinetic data of Scarpa and Graziotti [12] and Vinogradov and Scarpa [13] for the rat heart and liver mitochondria, respectively. The rate constants 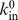 and 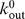 and the biophysical parameters *α*, *β*_e_, and *β*_x_ are arbitrarily chosen to satisfy the two kinetic and thermodynamic constraints of Eq. (S7) as well as to fit these data sets, as these parameters can not be accurately estimated from these data sets in which ΔΨ is constant. These extra-matrix Ca^2+^ dependent data sets, however, provide accurate estimates of the binding constants 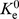 and 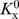. In the second step, with the values of 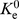 and 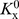 fixed, as estimated in the first step from the data of Vinogradov and Scarpa [13] for rat liver mitochondria, the remaining parameters 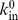, 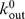, *α*, *β*_e_, and *β*_x_ are estimated from the ΔΨ dependent kinetic data of Wingrove et al. [16] for rat liver mitochondria, subject to the two kinetic and thermodynamic constraints of Eq. (A7). These ΔΨ dependent data provide accurate estimates of the biophysical parameters *α*, *β*_e_, and *β*_x_. In the final step, with the values of 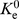, 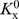, *α*, *β*_e_, and *β*_x_ fixed, as estimated from the first and second steps, the rate constants 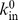 and 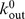 are estimated for the data sets of Scarpa and Graziotti [12] and Vinogradov and Scarpa [13], to allow these parameters to vary over data sets due to different experimental preparations (e.g., mitochondria from the rat heart vs. rat liver, presence of varying amount of Mg^2+^ in the experimental buffer).

Based on this model reparameterization, Case 2 provides multiple estimates of the kinetic parameters 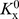 and 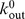 (see Table 1), all giving exactly the same fittings of the model to the data as for Case 1 (not shown, see below). In this case, the sensitivities of the least-square error to these parameters are extremely low, compared to the other parameters. The biophysical parameters were uniquely estimated as *α*_e_ = *α*_x_ = *α*≈0, *β*_e_≈0.113, and *β*_x_≈0.887. The new model fittings to the data for both the cases (Case 1 and Case 2) are exactly same as those shown in Figure 1 (A–C; upper panel), and hence are not shown here again; the new kinetic parameter estimates are also of comparable order of magnitudes for both the cases (see Table 1). Therefore, Case 2 is unidentifiable as a distinct case and is indistinguishable from Case 1. These model simulation analyses merely suggest that Case 2 in which the external and internal Ca^2+^ binding constants for the uniporter were assumed to be distinct (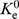
≠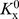 and 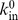
≠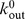) in our previous uniporter model [11] should not be accepted as a possible explanation for the observed ΔΨ dependency of uniporter-mediated mitochondrial Ca^2+^ uptake [16]. Alternatively, the external and internal Ca^2+^ binding constants for the uniporter may be equal (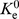 = 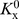 and 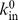 = 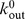).

**Table 1:** Reestimated parameter values for our previous models of mitochondrial Ca^2+^ uniporter with additional constraint: *α*_e_ = *α*_x_ = *α*≥0.

Parameters	Model 1		Model 2		References
	Case 1	Case 2	Case 1	Case 2	
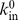	44.50.300.34	44.50.300.34	32.60.380.43	32.60.380.43	[16] [13] [12]
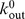	44.50.300.34	44.5×*m^2^*0.30×*m^2^*0.34×*m^2^*	32.60.380.43	32.6×*m^2^*0.38×*m^2^*0.43×*m^2^*	[16] [13] [12]
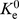	45.9×10^−6^45.9×10^−6^88.1×10^−6^	45.9×10^−6^45.9×10^−6^88.1×10^−6^	38.7×10^−6^38.7×10^−6^74.3×10^−6^	38.7×10^−6^38.7×10^−6^74.3×10^−6^	[16] [13] [12]
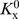	45.9×10^−6^45.9×10^−6^88.1×10^−6^	45.9×10^−6^×*m*45.9×10^−6^×*m*88.1×10^−6^×*m*	38.7×10^−6^38.7×10^−6^74.3×10^−6^	38.7×10^−6^×*m*38.7×10^−6^×*m*74.3×10^−6^×*m*	[16] [13] [12]
*α*_e_ = *α*_x_ = *α*	0.0	0.0	0.0	0.0	[12], [13], [16]
*β*_e_	0.113	0.113	0.113	0.113	[12], [13], [16]
*β*_x_	0.887	0.887	0.887	0.887	[12], [13], [16]

The rate constants 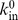 and 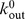 are redefined here as 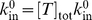 and 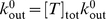 and are in the units of nmol/mg/s; the binding constants 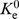 and 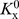 are in the units of molar. The kinetic and biophysical parameters satisfy the kinetic and thermodynamic constraints: 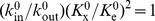 and 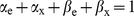 as well as the additional constraint: *α*_e_ = *α*_x_ = *α*≥0. The Case 1 corresponds to 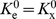 and 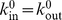, while the Case 2 corresponds to 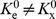 and 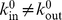. Note that *m* is an arbitrary number indicating the existence of multiple estimates of the kinetic parameters 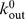 and 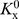 in Case 2 of the uniporter model.

**Figure 3:**
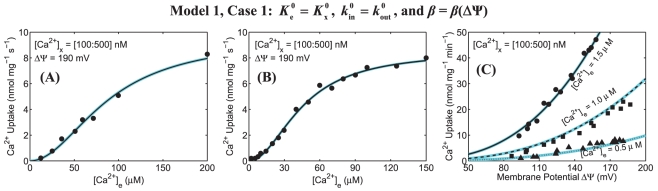
Predicted sensitivities (color maps) of mitochondrial Ca^2+^ uptake as functions of extra-matrix [Ca^2+^] and ΔΨ in response to variation in matrix [Ca^2+^] using our present alternate model of mitochondrial Ca^2+^ uniporter and their comparisons to the available experimental data (points). Simulations and fittings are shown only for Model 1 under Case 1 with the biophysical parameters *β*_e_ and *β*_x_ as functions of ΔΨ. Plot A shows the model simulations and fittings to the kinetic data of Scarpa and Graziotti [12] in which the initial rates of Ca^2+^ uptake were measured in respiring mitochondria isolated from rat hearts with varying levels of extra-matrix Ca^2+^. Plot B shows the model simulations and fittings to the kinetic data of Vinogradov and Scarpa [13] in which the initial rates of Ca^2+^ uptake were measured in respiring mitochondria isolated from rat livers with varying levels of extra-matrix Ca^2+^. For these analyses, ΔΨ was fixed at 190 mV, corresponding to state 2 respiration. Plot C shows the model simulations and fittings to the kinetic data of Wingrove et al. [16] in which the initial rates of Ca^2+^ uptake were measured as a function of ΔΨ in respiring mitochondria isolated from rat livers with three different levels of extra-matrix Ca^2+^ ([Ca^2+^]_e_ = 0.5 µM, 1.0 µM, and 1.5 µM). For sensitivity color maps, matrix [Ca^2+^] was varied from 100 nM to 500 nM. The black lines corresponding to the model fittings to the data are based on 250 nM of matrix [Ca^2+^]. The model uses the parameter values estimated based on the present uniporter model (Table 2).

**Figure 4:**
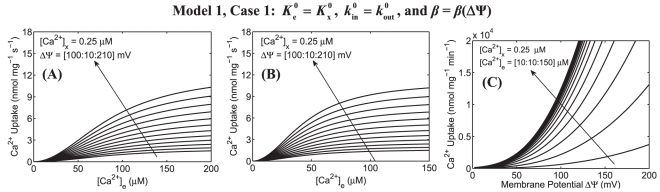
Predictions of mitochondrial Ca^2+^ uptake as a function of extra-matrix [Ca^2+^] for a range of ΔΨ and as a function ΔΨ for a range of extra-matrix [Ca^2+^] based on our present alternate model of mitochondrial Ca^2+^ uniporter. Simulations are shown only for Model 1 under Case 1 with the biophysical parameters *β*_e_ and *β*_x_ as functions of ΔΨ. Plot A shows the model predicted Ca^2+^ uptake in response to varying extra-matrix Ca^2+^ corresponding to the experimental protocol of Scarpa and Graziotti [12] for a range of ΔΨ. Plot B shows the model predicted Ca^2+^ uptake in response to varying extra-matrix Ca^2+^ corresponding to the experimental protocol of Vinogradov and Scarpa [13] for a range of ΔΨ. In these simulations, ΔΨ was varied from 100 mV to 210 mV and matrix [Ca^2+^] was fixed at 0.25 µM. Plot C shows the model predicted Ca^2+^ uptake as a function of ΔΨ corresponding to the experimental protocol of Wingrove et al. [16] with extra-matrix [Ca^2+^] ranging from 10 µM to 150 µM and matrix [Ca^2+^] fixed at 0.25 µM. The model uses the parameter values estimated based on the present uniporter model (Table 2).

The simulation analyses of mitochondrial Ca^2+^ uptake based on our present alternative model (Model 1) of mitochondrial Ca^2+^ uniporter, in which 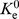 = 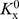 = 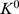, 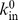 = 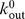 = 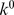, *α*_e_ = *α*_x_ = *α*, and *β*_e_ and *β*_x_ are functions of ΔΨ and *nH* (see Eq. 7), are shown in Figures 3 and 4. For model parameterization, a similar modular approach is used as described above. Specifically, the extra-matrix Ca^2+^ dependent kinetic data of Scarpa and coworkers [12], [13] are first used to get an estimate of the binding constant 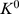 of Ca^2+^ to the uniporter with arbitrary values of 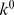, *α*, and *nH*. This estimated 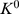 value is then fixed and the remaining three parameters (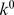, *α*, and *nH*) are estimated from the ΔΨ dependent kinetic data of Gunter and coworkers [16]. In the final step, with the estimated values 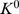, *α*, and *nH* fixed, the rate constant 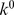 for the data of Scarpa and coworkers [12], [13] is estimated. This approach enables us to obtain a unique and robust set of parameters for our alternate model of the uniporter (see Table 2).

**Table 2:** Estimated parameter values for our present models of mitochondrial Ca^2+^ uniporter, in which 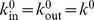, 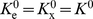, *α*_e_ = *α*_x_ = *α*≥0, and *β* = *β* (*nH*,ΔΨ).

Parameters	Model 1	Model 2	References
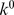	0.0159, 0.0142, 1.99	0.01972, 0.01775, 1.42	[12], [13], [16]
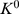	87.6, 45.6, 45.6	72.8, 37.9, 37.9	[12], [13], [16]
*α*	0.0	0.0	[12], [13], [16]
*nH*	2.65	2.65	[12], [13], [16]

The rate constants 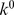 are in the units of nmol/mg/s and the binding constants 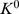 are in the units of micro-molar (µM). The biophysical parameters *β*_e_ and *β*_x_ are obtained as functions of *nH* and ΔΨ from Eq. (7).

Figure 3 depicts the model predicted sensitivities of Ca^2+^ fluxes via the uniporter (lines) as functions of extra-matrix [Ca^2+^] (ΔΨ = 190 mV) and ΔΨ ([Ca^2+^]_e_ = 0.5 µM, 1.0 µM, and 1.5 µM) with the variation in matrix [Ca^2+^] (100 nM to 500 nM) and their comparisons to the available experimental data [12], [13], [16] (points), obtained with matrix [Ca^2+^] = 250 nM. Figure 4 depicts the model predicted Ca^2+^ fluxes via the uniporter as a function of extra-matrix [Ca^2+^] for a range of ΔΨ (100 mV to 210 mV) and as a function of ΔΨ for a range of extra-matrix [Ca^2+^] (10 µM to 150 µM), obtained with matrix [Ca^2+^] = 250 nM. The left, middle, and right plots correspond to the model simulation analyses based on the experimental protocols of Scarpa and Graziotti [12], Vinogradov and Scarpa [13], and Wingrove et al. [16], respectively.

The model simulation analyses in Figure 3 demonstrate that our present alternative model of the uniporter is able to match all the available experimental data [12], [13], [16] on the kinetics of both the extra-matrix Ca^2+^ and ΔΨ dependencies of mitochondrial Ca^2+^ uptake via the uniporter in the entire ranges of extra-matrix [Ca^2+^] and ΔΨ for which data were available. In addition, this alternate uniporter model is insensitive to variation in matrix [Ca^2+^], making the model physiologically plausible. This characteristic of the model helps provide unique and accurate estimates of the model parameters with different matrix [Ca^2+^]. It is observed from Figure 4 that the Ca^2+^ uptake profiles, obtained from the present alternate uniporter model, do not have stiff gradients with respect to ΔΨ, and reach saturation for a higher level of extra-matrix [Ca^2+^], comparable to that obtained under Case 1 of our previous uniporter model (see Figure 2 (A–C, upper panel), but unlike to that obtained under Case 2 of our previous uniporter model (Figure 2 (D–F), lower panel). The estimates of the Michaelis-Menten kinetic parameters 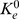 and 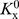 based on the previous and present models of the uniporter obtained with the constraint: *α*_e_ = *α*_x_ = *α*≥0 are of comparable order of magnitudes (compare 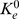 and 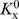 from Table 1 vs. Table 2).

The fittings of Model 1 (Case 1) to the extra-matrix Ca^2+^ dependent data of Scarpa and Graziotti [12] from cardiac mitochondria and Vinogradov and Scarpa [13] from liver mitochondria provides the estimates 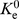 = 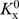 = 87.6 µM and 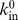 = 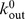 = 0.0159 nmol/mg/sec and 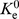 = 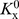 = 45.6 µM and 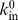 = 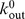 = 0.0142 nmol/mg/sec, respectively (see Table 2). These differences in the estimates of the kinetic parameters may be attributed towards the fact that the data are from two different mitochondrial preparations and two different experimental protocols (e.g., the differences in the amount of Mg^2+^ present in the two experimental buffer mediums, which is known to compete with Ca^2+^ for transport into mitochondria via the uniporter, and hence inhibits mitochondrial Ca^2+^ uptake [12]–[15], [17]). Analysis of these kinetic data with Model 2 (Case 1) showed that the model parameter values are readjusted to provide similar fits of the model to the three independent experimental data sets (see Table 2).

**Figure 5:**
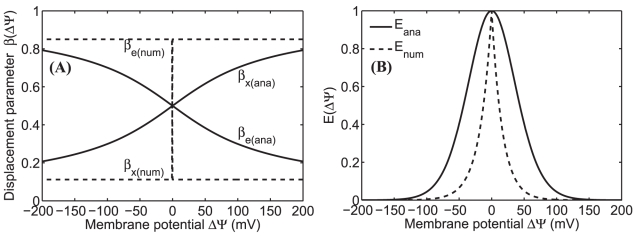
Variation of biophysical parameters β_e_ and β_x_ as functions of ΔΨ and the corresponding ΔΨ dependent factors in the uniporter flux expressions as functions of β_e_ and β_x_. The solid lines are based on our present model of the uniporter in which β_e_ and β_x_ are analytical functions of ΔΨ, while the dotted lines are based on our previous model of the uniporter in which β_e_ and β_x_ are constants and are numerically estimated.

The ΔΨ dependencies of the biophysical parameters *β*_e_ and *β*_x_ that characterize the ΔΨ dependent factor E(ΔΨ) in the uniporter flux expressions (see Eqs. S8, 5, and 6), and hence the ΔΨ dependency of mitochondrial Ca^2+^ uptake via the uniporter [16], are demonstrated in Figure 5. The solid lines are based on our present alternate model of the uniporter in which *β*_e_ and *β*_x_ are analytical functions of ΔΨ (Eq. 7), while the dotted lines are based on our previous model of the uniporter [11] in which *β*_e_ and *β*_x_ are constants and are numerically estimated. The results show that *β*_e_ and *β*_x_ based on the previous model abruptly change their respective values at ΔΨ = 0, resulting in non-differentiability of the factor E(ΔΨ) at ΔΨ = 0. In contrast, *β*_e_, *β*_x_, and E(ΔΨ) based on the present model are smooth functions of ΔΨ. The ΔΨ dependent factor E(ΔΨ) in the present model has a larger spread, resulting in a better fit of the model to the ΔΨ dependent data [16]. Also the two ΔΨ dependent factors differ in the domain ΔΨ≤120 mV, describing the discrepancy of the fitting of the two models to the ΔΨ dependent data [16] in this domain.

## Discussion

The major contributions of the present paper lies in the improvements of our previous biophysical model of mitochondrial Ca^2+^ uniporter [11], which is the primary influx pathway for Ca^2+^ into energized (respiring) mitochondria, and hence plays an important role in mitochondrial Ca^2+^ homeostasis. Specifically, the present paper provides an alternate (improved) biophysical model of the uniporter that overcomes the limitations of our previous uniporter model by mechanistically recharacterizing the membrane potential (ΔΨ) dependency of mitochondrial Ca^2+^ uptake via the uniporter. This alternate uniporter model is able to accurately characterize the possible mechanisms of both the extra-matrix Ca^2+^ and ΔΨ dependencies of the uniporter-mediated mitochondrial Ca^2+^ uptake [12], [13], [16]. This model along with our recently developed biophysical model of mitochondrial Na^+^/Ca^2+^ exchanger [27] is important in developing mechanistic, integrated models of mitochondrial bioenergetics and Ca^2+^ handling that can be helpful in understanding the mechanisms by which Ca^2+^ plays a role in mediating signaling pathways between cytosol and mitochondria and modulating mitochondrial energy metabolism in health and disease.

Our previous model of the uniporter [11] was developed based on a multi-state catalytic binding and interconversion mechanism (Michaelis-Menten kinetics) for carrier-mediated facilitated transport [22], [23], combined with Eyring's free-energy barrier theory for interconversion and electrodiffusion (Ca^2+^ translocation) [22], [24]–[26]. The model also accounts for possible allosteric cooperative binding of Ca^2+^ to the uniporter, as depicted experimentally [12], [13]. The model was parameterized based on comparisons of model simulated outputs under various kinetic assumptions (Model 1 or Model 2: fully or partial cooperative binding of Ca^2+^ to the uniporter; Case 1 or Case 2: external and internal Ca^2+^ binding constants for the uniporter are equal or distinct) to several independent experimental data sets from the literature on the kinetics of Ca^2+^ fluxes via the uniporter [12], [13], [16], measured in suspensions of respiring mitochondria purified from rat hearts and rat livers under varying experimental conditions. The model was able to adequately describe the extra-matrix Ca^2+^ dependent data of Scarpa and coworkers [12], [13] as well as the ΔΨ dependent data of Gunter and coworkers [16] with the assumption that the dissociation constants associated with the binding of external and internal Ca^2+^ to the uniporter are distinct (Case 2). Therefore, the mechanistic formulation, thermodynamic feasibility, and ability to describe a large number of independent experimental data sets are some of the notable features of the model [11], compared to the previous models of the uniporter [19]–[21]. Since the present alternate uniporter model is developed from the previous uniporter model by exclusively reformulating the ΔΨ dependencies of the rate constants of Ca^2+^ translocation, the present model has all the characteristics of the previous model.

In the experimental studies of Scarpa and colleagues [12], [13] and Gunter and colleagues [16], matrix [Ca^2+^] was not measured. Our two variant models of the uniporter under two different cases [11] were parameterized entirely based on these experimental data [12], [13], [16] with a fixed matrix [Ca^2+^] of 250 nM. Although both the variant models under Case 2 were able to satisfactorily explain all the available experimental data with suitable model perturbations as provided by the experimental protocols, it was not realized whether physiological variation of matrix [Ca^2+^], as observed in the intact myocyte, will have any appreciable effect on the estimates of model parameters and model predicted trans-matrix Ca^2+^ fluxes via the uniporter. The present study provides a quantitative reevaluation of the previous uniporter model [11] to test the robustness of the estimates of model parameters and model predictions subject to physiologically reasonable variation in matrix [Ca^2+^] ranging from 100 nM to 500 nM. Based on this model simulation analysis, it is found that the two variant model predictions under Case 2 are highly sensitive to variation in matrix [Ca^2+^] (Figure 1 (D–F): lower panel), signifying that the model parameter estimates under Case 2 would vary considerably to variation in matrix [Ca^2+^], and hence can not be robust. In addition, Case 2 was associated negative estimates of the biophysical parameter *α*_x_ (with *α*_e_ = 0 fixed) (Table S1), attributing to the high sensitivities of the model predictions to variation in matrix [Ca^2+^] (Figure 1(D–F): lower panel) and stiff gradients of Ca^2+^ uptake profiles to variation in ΔΨ (Figure 2 (D–F): lower panel). The reparameterization of the model subject to the constraint: *α*_e_ = *α*_x_ = *α*≥0 showed that Case 2 is unidentifiable as a distinct case and is indistinguishable from Case 1.

In summary, the reevaluation of our previous model of mitochondrial Ca^2+^ uniporter [11] simply suggest that Case 2 in which the external and internal Ca^2+^ binding constants for the uniporter were assumed distinct (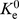
≠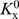 and 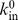
≠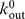) is unacceptable as a possible explanation for the observed ΔΨ dependency of mitochondrial Ca^2+^ uptake via the uniporter [16]. On the other hand, the external and internal Ca^2+^ binding constants for the uniporter should be equal (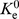 = 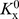 and 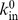 = 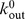) (Table 2), and the biophysical parameters *β*_e_ and *β*_x_ associated with the free-energy barrier of Ca^2+^ translocation via the uniporter should be dependent on ΔΨ (Eq. B7; Figure 5). The alternate uniporter model based on this revised ΔΨ dependent formulation for *β*_e_ and *β*_x_ is shown to satisfactorily reproduce all the available experimental data [12], [13], [16] on the kinetics of both the extra-matrix Ca^2+^ and ΔΨ dependencies of mitochondrial Ca^2+^ uptake via the uniporter in the entire ranges of extra-matrix [Ca^2+^] and ΔΨ for which data were available (Figure 3). In addition, the model is insensitive to variation in matrix [Ca^2+^], predicting relatively stable physiological operation of the uniporter.

Recently, mitochondria have been recognized as one of the key organelles that actively involves in physiological Ca^2+^ signaling [6], [8], [28]. Its ability to buffer Ca^2+^ in distinct region of the cells and maintain spatial Ca^2+^ concentration low even under strong global Ca^2+^ mobilization upon cell stimulation is critical for the Ca^2+^ sensitive signal transduction within the cell [29]. It is also evident that, mitochondria can potentially modulate the nature of intracellular Ca^2+^ oscillations and waves, generated by the Ca^2+^ release from the endoplasmic reticulum [30], [31]. While numerous efforts have been made to model intracellular Ca^2+^ oscillations, the mitochondrial Ca^2+^ uptake has also been shown to influence significantly the nature of Ca^2+^ oscillations [32]. Furthermore, in many cell types, the respiring mitochondria remain critical for the activity and maintenance of capacitive Ca^2+^ entry [33], [34]. For example, in a recent study it has been shown that, the mitochondrial Ca^2+^ uptake has considerable effect on the STIM1-Orai1-dependent store operated Ca^2+^ entry into endothelial cells [35]. In this context, the present model of mitochondrial Ca^2+^ uniporter will be crucial for developing an integrated model of intracellular and mitochondrial Ca^2+^ handling which can be helpful in understanding many aspects of signal transduction mechanisms.

## Materials and Methods

### Alternate Model of Mitochondrial Ca^2+^ Uniporter

This section provides the derivation of the present alternate (improved) biophysical model of mitochondrial Ca^2+^ uniporter that accurately characterizes the ΔΨ dependency of mitochondrial Ca^2+^ uptake via the uniporter [16] under Case 1 (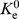 = 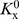 and 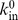 = 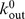) and overcomes the limitations of our previous biophysical model of the uniporter [11]. Specifically, to accurately characterize this ΔΨ dependency under Case 1, we reformulate the ΔΨ dependencies of the rate constants *k*_in_ and *k*_out_ of Ca^2+^ translocation (Eq. S5, Materials S1) in our previous uniporter model [11] by exclusively redefining the biophysical parameters *β*_e_ and *β*_x_ associated with the free-energy barrier of Ca^2+^ translocation based on a generalized, non-linear Goldman-Hodgkin-Katz (GHK) formulation. The expressions for the binding constants *K*_e_ and *K*_x_ and the equilibrium constants *K*_eq_ remain the same as in Eqs. (S4) and (S6) of Materials S1. Therefore, the present alternative uniporter model has all the characteristics of our previous uniporter model [11]. We also illustrate that this uniporter model is able to characterize the possible mechanisms of both the extra-matrix Ca^2+^ and ΔΨ dependencies of the uniporter-mediated mitochondrial Ca^2+^ uptake [12], [13], [16] under Case 1. Furthermore, this uniporter model is insensitive to variation in matrix [Ca^2+^], making the model physiologically plausible.

Based on a generalized, non-linear GHK formulation, the rate constants *k*_in_ and *k*_out_ of Ca^2+^ translocation via the uniporter (see Eq. S5) can be expressed in the following form [1], [9]:
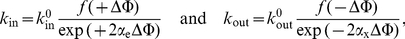
(1)where 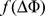 is an unknown non-linear function to be determined. Substituting Eq. (1) for *k*_in_ and *k*_out_ and Eq. (S4) for *K*_e_ and *K*_x_ into Eqs. (S1–S2), the uniporter flux expression is reduced to
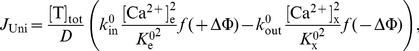
(2)where *D = D*_1_ for Model 1 (Eq. S9a) and *D = D*_2_ for Model 2 (Eq. S9b). In order to derive the functional form of 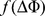, we consider the equilibrium condition for trans-membrane Ca^2+^ transport via the uniporter 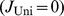, which in combination with Eqs. (S3) and (S6) gives:
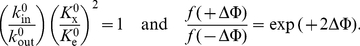
(3)Under Case 1 (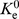 = 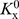 and 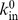 = 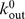), the kinetic constraint of Eq. (3) is automatically satisfied. However, the thermodynamic constraint of Eq. (3) provides multiple solutions for 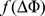. The general solution that satisfies the equilibrium condition for passive Ca^2+^ transport via the uniporter in the absence of ΔΦ 
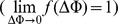
 is given by
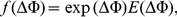
(4a)where
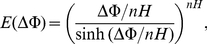
(4b)is an even function: 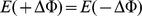; 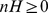 is an arbitrary number to be determined. Thus, the unknown function 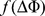 is fully characterized by only one unknown parameter *nH*, and hence the two rate constants *k*_in_ and *k*_out_ in Eq. (1) are fully characterized by only three unknown parameters 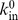, 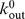 and *nH*, in contrast to four unknown parameters 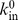, 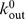, *β*_e_ and *β*_x_ in the previous formulation (see Materials S1). In standard GHK formulation (linear, constant field-type approximation) for interconversion and electrodiffusion of the uniporter-2Ca^2+^ complex, *nH* = 1, 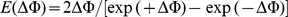, and 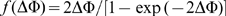 [1], [9]. Substituting Eq. (4) into Eq. (2), the uniporter flux expression can be expressed as
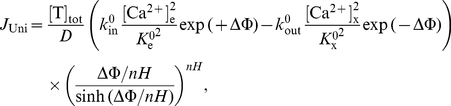
(5)where *D = D*_1_ for Model 1 (Eq. S9a) and *D = D*_2_ for Model 2 (Eq. S9b). By comparing the present uniporter model (Eq. 5) with the previous uniporter model (Eq. S8), we obtain the following functional relationship between the biophysical parameters *α*_e_, *α*_x_, *β*_e_, *β*_x_, and *nH*:
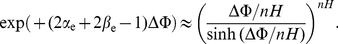
(6)Using Eq. (6) along with the thermodynamic constraint: 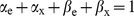, the biophysical parameters *β*_e_ and *β*_x_ can be expressed in terms of ΔΦ as
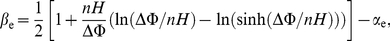
(7a)

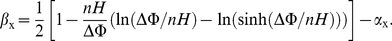
(7b)Therefore, the biophysical parameters *β*_e_ and *β*_x_ in the present model of the uniporter become functions of ΔΨ, compared to the previous model of the uniporter [11], in which *β*_e_ and *β*_x_ were constant with respect to ΔΨ. Furthermore, both the parameters are characterized by only one unknown parameter *nH*.

#### Model Parameterization

Both the kinetic models of the uniporter are characterized by seven unknown parameters: 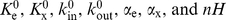. These parameters are estimated based on the experimental data of Scarpa and coworkers [12], [13] and Gunter and coworkers [16] on the kinetics of Ca^2+^ fluxes via the uniporter subject to the constraints: 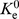 = 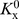 = 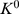, 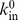 = 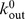 = 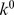, and *α*_e_ = *α*_x_ = *α*≥0 (Case 1). Thus, only four unknown parameters 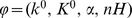 are estimated for both the uniporter models. A least-squares estimation technique is used in multiple steps to fit the model simulated outputs to the experimental data.
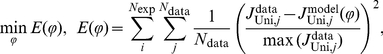
(8)where *N*_exp_ is the number of experiments and *N*_data_ is the number of data points in a particular experiment, 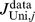 are the experimental data on Ca^2+^ uptakes and 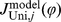 are the corresponding model simulated outputs which depend on the model parameters 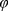, and 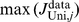 is the maximum value of 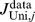. A MATLAB function optimizer FMINCON is used to minimize the mean residual error *E*(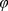) to estimate the model parameters 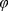. The accuracy and robustness of the model fitting to the data for a particular uniporter model is assessed based on the value of the mean residual error *E*(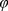) and its sensitivities to perturbations in the optimal parameter estimates.

## Supporting Information

Materials S1 Supporting materials that briefly describe the previous models of mitochondrial Ca^2+^ uniporter.
(0.14 MB DOC)

Table S1 Estimated parameter values in the previous models of mitochondrial Ca^2+^ uniporter.
(0.09 MB DOC)
