# Supplementary material for: Characterization of Membrane Potential Dependency of Mitochondrial Ca2+ Uptake by an Improved Biophysical Model of Mitochondrial Ca2+ Uniporter
Source: PLoS One. 2010 Oct 8;5(10):e13278. doi: 10.1371/journal.pone.0013278 (PMC2951907; doi:10.1371/journal.pone.0013278)
Supplement: Materials S1 — Supporting materials that briefly describe the previous models of mitochondrial Ca2+ uniporter. (0.14 MB DOC) [file pone.0013278.s001.doc]

## Materials S1: Previous Model of Mitochondrial Ca^2+^ Uniporter

This supporting materials provides a brief description of our previous biophysical model of mitochondrial Ca^2+^ uniporter [1] that adequately describes the available experimental data on the kinetics of mitochondrial Ca^2+^ uptake [2-4]. This model was developed based on a Michaelis-Menten kinetics for multi-state catalytic binding and interconversion mechanism associated with carrier-mediated facilitated transport [5,6], combined with Eyring’s free energy-barrier theory for absolute reaction rates associated with interconversion and electrodiffusion (Ca^2+^ translocation) [5,7-9] (see Figures 1 and 2 of Dash et al. [1]). The model also accounts for a possible mechanism that assumes allosteric, cooperative binding of Ca^2+^ to the uniporter, as observed experimentally [2,3]. The associated kinetic flux expressions are given by

$J_{\text{Uni}}=\frac{[\text{T}]_{\text{tot}}}{D}\left(k_{\text{in}}\frac{[\text{Ca}^{\text{2+}}\text{]}_{\text{e}}^{2}}{K_{\text{e}}^{2}}-k_{\text{out}}\frac{[\text{Ca}^{\text{2+}}\text{]}_{\text{x}}^{2}}{K_{\text{x}}^{2}}\right),$ (S1)

where

$D=D_{1}=1+\frac{[\text{Ca}^{\text{2+}}\text{]}_{\text{e}}^{2}}{K_{\text{e}}^{2}}+\frac{[\text{Ca}^{\text{2+}}\text{]}_{\text{x}}^{2}}{K_{\text{x}}^{2}}\text{ (Model 1),}$ (S2a)

$D=D_{2}=1+\frac{[\text{Ca}^{\text{2+}}\text{]}_{\text{e}}}{K_{\text{e}}}+\frac{[\text{Ca}^{\text{2+}}\text{]}_{\text{x}}}{K_{\text{x}}}+\frac{[\text{Ca}^{\text{2+}}\text{]}_{\text{e}}\text{[Ca}^{\text{2+}}\text{]}_{\text{x}}}{K_{\text{e}}K_{\text{x}}}+\frac{[\text{Ca}^{\text{2+}}\text{]}_{\text{e}}^{2}}{K_{\text{e}}^{2}}+\frac{[\text{Ca}^{\text{2+}}\text{]}_{\text{x}}^{2}}{K_{\text{x}}^{2}} \text{(Model 2).}$ (S2b)

Here *K*_e_ and *K*_x_ are the dissociation constants associated with the binding of extra-matrix and matrix Ca^2+^ to the uniporter (T); *k*_in_ and *k*_out_ are the rate constants associated with the translocation of Ca^2+^ via the uniporter; [T]_tot_ represents the total uniporter concentration. Model 1 depicts the full cooperative binding, while Model 2 depicts the partial cooperative binding of Ca^2+^ to the uniporter. The kinetic parameters *K*_e_, *K*_x_, *k*_in_ and *k*_out_ are constrained by

$\frac{k_{\text{in}}K_{\text{x}}^{2}}{k_{\text{out}}K_{\text{e}}^{2}}=\frac{[\text{Ca}^{\text{2+}}\text{]}_{\text{x,eq}}^{2}}{[\text{Ca}^{\text{2+}}\text{]}_{\text{e,eq}}^{2}}=K_{\text{eq}}.$ (S3)

where *K*_eq_ is the equilibrium constant for trans-membrane Ca^2+^ transport defined below.

**ΔΨ Dependencies of the Kinetic Parameters:** The electrostatic field of the charged membrane influences both the binding stages of Ca^2+^ to the uniporter and translocation stages of Ca^2+^ via the uniporter. In our previous uniporter model [1], these interactions are described via dependencies of the kinetic parameters *K*_eq_, *K*_e_, *K*_x_, *k*_in_ and *k*_out_ on the membrane potential ΔΨ, which were derived based on biophysical principles and well-known laws of thermodynamics, electrostatics, and superposition:

$K_{\text{e}}=K_{\text{e}}^{0}\exp(-\alpha_{\text{e}}\Delta\Phi)\text{ and }K_{\text{x}}=K_{\text{x}}^{0}\exp(+\alpha_{\text{x}}\Delta\Phi),$ (S4)

$k_{\text{in}}=k_{\text{in}}^{0}\exp(+2\beta_{\text{e}}\Delta\Phi)\text{ and }k_{\text{out}}=k_{\text{out}}^{0}\exp(-2\beta_{\text{x}}\Delta\Phi),$ (S5)

$K_{\text{eq}}=\exp(+2\Delta\Phi),\text{ where }\Delta\Phi=Z_{\text{Ca}}F\Delta\Psi/RT.$ (S6)

Here *Z*_Ca_ = 2 is the valence of Ca^2+^; *F*, *R*, and *T* denote the Faraday’s constant, ideal gas constant, and absolute temperature, respectively (*F* = 96484.6 J/mol/V and *R* = 8.3145 J/mol/K); *α*_e_ (*α*_x_) is the ratio of the potential difference between Ca^2+^ bound at the site of the uniporter facing the cytosolic (matrix) side of the IMM and Ca^2+^ in the bulk phase to the total membrane potential ΔΨ; *β*_e_ (*β*_x_) represents the displacement of cytosolic (matrix) Ca^2+^ from the coordinate of maximum potential barrier. Thus, the two dissociation constants *K*_e_ and *K*_x_ were fully characterized by four unknown parameters $K_{\text{e}}^{0}$, $K_{\text{x}}^{0}$, *α*_e_ and *α*_x_, and the two rate constants *k*_in_ and *k*_out_ were fully characterized by four unknown parameters $k_{\text{in}}^{0}$, $k_{\text{out}}^{0}$, *β*_e_ and *β*_x_. By substituting Eq. (S4) for *K*_e_ and *K*_x_, Eq. (S5) for *k*_in_ and *k*_out_, and Eq. (S6) for *K*_eq_ into Eq. (S3), the following kinetic and thermodynamic constraints are obtained:

$\left(\frac{k_{\text{in}}^{0}}{k_{\text{out}}^{0}}\right)\left(\frac{K_{\text{x}}^{0}}{K_{\text{e}}^{0}}\right)^{2}=1\text{ and }\alpha_{\text{e}}+\alpha_{\text{x}}+\beta_{\text{e}}+\beta_{\text{x}}=1.$ (S7)

**Reduced Flux Expressions:** Substituting Eq. (S4) for *K*_e_ and *K*_x_ and Eq. (S5) for *k*_in_ and *k*_out_ into Eqs. (S1-S2), and using the kinetic and thermodynamic constraints of Eq. (S7), the uniporter flux expressions are reduced to

(S8)

where

(S9a)

(S9b)

**Model Parameterization and Simulations:** Both the kinetic models of the uniporter are characterized by eight unknown parameters: $K_{\text{e}}^{0}$, $K_{\text{x}}^{0}$, $k_{\text{in}}^{0}$, $k_{\text{out}}^{0}$, *α*_e_, *α*_x_, *β*_e_, and *β*_x_. The number of parameters for estimation was reduced to six by using the two constraints of Eq (S7). In addition, the parameter estimation procedure was carried out under two different kinetic assumptions: *α*_e_ = 0, $K_{\text{e}}^{0}$=$K_{\text{x}}^{0}$, and $k_{\text{in}}^{0}$=$k_{\text{out}}^{0}$ (Case 1) and *α*_e_ = 0, $K_{\text{e}}^{0}$≠$K_{\text{x}}^{0}$, and $k_{\text{in}}^{0}$≠$k_{\text{out}}^{0}$ (Case 2) on the binding of extra-matrix and matrix Ca^2+^ to the uniporter leading to the formation of four variant kinetic models of the uniporter. The number of parameters for estimation was reduced to four in Case 1 and five in Case 2. These parameters were estimated based on the experimental data of Scarpa and colleagues [2,3] and Gunter and colleagues [4] on the kinetics of Ca^2+^ fluxes via the uniporter, measured in suspensions of respiring mitochondria purified from rat hearts and rat livers under varying experimental conditions (varying extra-matrix [Ca^2+^] and varying ΔΨ). The estimated parameter values are summarized in Table S1.

Both the models under both the cases were able to adequately describe the extra-matrix Ca^2+^ dependent data of Scarpa and coworkers [2,3]. However, the two different Ca^2+^ binding cases (Case 1 and Case 2) provided two significantly different predictions of the ΔΨ dependent data of Gunter and coworkers [4], particularly in the range ΔΨ ≤ 120 mV. While the models under Case 1 ($K_{\text{e}}^{0}$=$K_{\text{x}}^{0}$ and $k_{\text{in}}^{0}$=$k_{\text{out}}^{0}$) were not able to simulate the ΔΨ dependent data in the range ΔΨ ≤ 120 mV, the models under Case 2 ($K_{\text{e}}^{0}$≠$K_{\text{x}}^{0}$ and $k_{\text{in}}^{0}$≠$k_{\text{out}}^{0}$) were able to adequately reproduce the ΔΨ dependent data in the entire ΔΨ range for which data were available. Based on these kinetic analyses, Case 2 was hypothesized as the most feasible scenario responsible for the observed ΔΨ dependency of mitochondrial Ca^2+^ uptake via the uniporter. In this case, the parameter estimates had the trends: *α*_x_ < 0, $K_{\text{e}}^{0}$ >> $K_{\text{x}}^{0}$, and $k_{\text{in}}^{0}$ << $k_{\text{out}}^{0}$ (see Table S1).

# References

1. Dash RK, Qi F, Beard DA (2009) A biophysically based mathematical model for the kinetics of mitochondrial calcium uniporter. Biophys J 96: 1318-1332.

2. Scarpa A, Graziotti P (1973) Mechanisms for intracellular calcium regulation in heart. I. Stopped-flow measurements of Ca^2+^ uptake by cardiac mitochondria. J Gen Physiol 62: 756-772.

3. Vinogradov A, Scarpa A (1973) The initial velocities of calcium uptake by rat liver mitochondria. J Biol Chem 248: 5527-5531.

4. Wingrove DE, Amatruda JM, Gunter TE (1984) Glucagon effects on the membrane potential and calcium uptake rate of rat liver mitochondria. J Biol Chem 259: 9390-9394.

5. Keener JP, Sneyd J (1998) Mathematical Physiology. New York: Springer. xx 766 p. p.

6. Fall CP, Wagner J, Marland E, editors (2002) Computational Cell Biology. New York: Springer. xx, 468 p. p.

7. Woodbury JW (1971) Eyring rate theory model of the current-voltage relationship of ion channels in excitable membranes. In: Hirschfelder J, editor. Chemical Dynamics: Papers in Honor of Henry Eyring. New York: John Wiley and Sons Inc.

8. Lauger P (1973) Ion transport through pores: a rate-theory analysis. Biochim Biophys Acta 311: 423-441.

9. Lauger P, Neumcke B (1973) Theoretical analysis of ion conductance in lipid bilayer membranes. Membranes 2: 1-59.
