# Supplementary material for: Characterization of Membrane Potential Dependency of Mitochondrial Ca2+ Uptake by an Improved Biophysical Model of Mitochondrial Ca2+ Uniporter
Source: PLoS One. 2010 Oct 8;5(10):e13278. doi: 10.1371/journal.pone.0013278 (PMC2951907; doi:10.1371/journal.pone.0013278)
Supplement: Table S1 — Estimated parameter values in the previous models of mitochondrial Ca2+ uniporter. (0.09 MB DOC) [file pone.0013278.s002.doc]

**Table S1: Estimated parameter values in the previous models of mitochondrial Ca^2+^ uni**porter.

| Parameters | Model 1 | | Model 2 | | References  (see Materials S1) |
| --- | --- | --- | --- | --- | --- |
|  | Case 1 | Case 2 | Case 1 | Case 2 |  |
| $k_{\text{in}}^{0}$ | 49.6  0.32  0.354 | 0.55  3.55×10^–3^  3.986×10^–3^ | 36.0  0.40  0.45 | 0.5  5.56×10^–3^  6.76×10^–3^ | [4]  [3]  [2] |
| $k_{\text{out}}^{0}$ | 49.6  0.32  0.354 | 8.5×10^–3^  54.84×10^–6^  61.59×10^–6^ | 36.0  0.40  0.45 | 0.028  311.1×10^–6^  378.4×10^–6^ | [4]  [3]  [2] |
| $K_{\text{e}}^{0}$ | 48×10^–6^  48×10^–6^  90×10^–6^ | 15×10^–6^  15×10^–6^  78.75×10^–6^ | 40×10^–6^  40×10^–6^  75×10^–6^ | 12×10^–6^  12×10^–6^  63×10^–6^ | [4]  [3]  [2] |
| $K_{\text{x}}^{0}$ | 48×10^–6^  48×10^–6^  90×10^–6^ | 1.865×10^–6^  1.865×10^–6^  9.79×10^–6^ | 40×10^–6^  40×10^–6^  75×10^–6^ | 2.84×10^–6^  2.84×10^–6^  14.91×10^–6^ | [4]  [3]  [2] |
| *α*_e_ | 0 | 0 | 0 | 0 | [2-4] |
| *α*_x_ | 0.038 | –0.214 | 0.038 | –0.239 | [2-4] |
| *β*_e_ | 0.112 | 0.264 | 0.112 | 0.259 | [2-4] |
| *β*_x_ | 0.85 | 0.95 | 0.85 | 0.98 | [2-4] |

The rate constants $k_{\text{in}}^{0}$ and $k_{\text{out}}^{0}$ are redefined here as $k_{\text{in}}^{0}=[T]_{\text{tot}}k_{\text{in}}^{0}$ and $k_{\text{out}}^{0}=[T]_{\text{tot}}k_{\text{out}}^{0}$ and are in the units of nmol/mg/s; the binding constants $K_{\text{e}}^{0}$ and $K_{\text{x}}^{0}$ are in the units of molar. The kinetic and biophysical parameters satisfy the kinetic and thermodynamic constraints: $(k_{\text{in}}^{0}/k_{\text{out}}^{0})(K_{\text{x}}^{0}/K_{\text{e}}^{0})^{2}=1$ and $\alpha_{\text{e}}+\alpha_{\text{x}}+\beta_{\text{e}}+\beta_{\text{x}}=1$. The Case 1 corresponds to $K_{\text{e}}^{0}=K_{\text{x}}^{0}$ and $k_{\text{in}}^{0}=k_{\text{out}}^{0}$, while the Case 2 corresponds to $K_{\text{e}}^{0}\neq K_{\text{x}}^{0}$ and $k_{\text{in}}^{0}\neq k_{\text{out}}^{0}$.
